# Effect of Instant Controlled Pressure Drop (DIC) on Polyphenols, Flavonoids and Antioxidant Capacity of Green Lentils (*Lens culinaris*)

**DOI:** 10.3390/molecules28104119

**Published:** 2023-05-16

**Authors:** Mario Adrian Tienda-Vazquez, Rocío Daniela Soto-Castro, Oscar Carrasco-Morales, Carmen Téllez-Pérez, Roberto Parra-Saldívar, Maritza Alonzo-Macías, Anaberta Cardador-Martínez

**Affiliations:** 1 Tecnologico de Monterrey, Escuela de Ingeniería y Ciencias, Epigmenio González 500, San Pablo 76130, Querétaro, Mexico; marioadriantiendavazquez@gmail.com (M.A.T.-V.); a01701721@tec.mx (O.C.-M.); ctellezperez@gmail.com (C.T.-P.); malonzoma@tec.mx (M.A.-M.); 2 Tecnologico de Monterrey, Centro de Biotecnologia FEMSA, School of Engineering and Sciences, Avenida Eugenio Garza Sada 2501, Monterrey 64849, Nuevo León, Mexico; a01706104@tec.mx (R.D.S.-C.); r.parra@tec.mx (R.P.-S.); 3 Laboratory of Engineering Science for Environment LaSIE-UMR-CNRS 7356, Eco-Intensification of Agro-Industrial Eco-Processes, La Rochelle University, 17042 La Rochelle, France

**Keywords:** DIC, DPPH, TEAC, Folin–Ciocalteu, HPLC, lentils, phenols

## Abstract

Instant controlled pressure drop (DIC) is one of the emerging technologies in food processing; it can be used for drying, freezing and the extraction of bioactive molecules without damaging their properties. Legumes, such as lentils, are one of the most consumed foods in the world; however, they are mainly cooked by boiling, which causes the loss of antioxidant compounds. This work evaluated the effect of 13 different DIC treatments (with pressure ranges of 0.1–0.7 MPa and times of 30–240 s) on the content of polyphenols (Folin–Ciocalteu and High Performance Liquid Chromatography HPLC) and flavonoids (2-aminoethyl diphenylborinate) as well as the antioxidant activity (DPPH and TEAC) of green lentils. The DIC 11 treatment (0.1 MPa, 135 s) obtained the best release of polyphenols, which in turn are related to antioxidant capacity. The abiotic stress generated by DIC could lead to the breakdown of the cell wall structure, which favors the availability of antioxidant compounds. Finally, the most efficient conditions for DIC to promote the release of phenolic compounds and maintain antioxidant capacity were found under low pressures (<0.1 MPa) and short times (<160 s).

## 1. Introduction

Thanks to their richness in macronutrients (e.g., proteins) and bioactive compounds (antioxidants), legumes such as peas, beans and lentils are among the most consumed foods in the world [1,2]. Mexico is one of the countries where legumes are fundamental in the traditional diet. Specifically, lentils (*Lens culinaris*) are consumed by 70% of Mexican adults and present a significant content of protein (21–31%), carbohydrates (62–69%) mainly made up of starch, and different antioxidants, such as polyphenols, which are well known by their relevant use against degenerative diseases, such as cancer and cardiovascular diseases [3,4,5,6]. Moreover, there are different varieties of colors between types of lentils, ranging from green, red, brown and black [1,7]. Additionally, according to some studies, green lentils (GL) are the legumes with the highest reported antioxidant content when evaluated with different antioxidant capacity measurement methods; this has been attributed to their great variety in secondary metabolites [8]. However, as with most legumes, lentils are mainly cooked by boiling, which causes the loss of bioactive compounds [8]. In this respect, instant controlled pressure drop technology (DIC: *Détente Instantanée Contrôlée*) is a promising emerging technology to preserve the macro- and micronutrients of lentils. DIC technology is a thermomechanical treatment that causes the expansion of the biological matrix by applying high pressures with saturated steam (generally between 100 and 700 kPa) during short periods (a few seconds), followed by an instant pressure drop through a vacuum (between 5 and 10 kPa) [9]. This causes an expansion of the food matrix, releasing multiple bioactive compounds without damaging their properties [9,10,11,12,13]. In this respect, the present study aims to analyze the effect of DIC treatment (with different pressures and times) in the polyphenols and flavonoids as well as the antioxidant capacity of green lentils.

## 2. Results and Discussion

### 2.1. Effect of DIC Treatments on Total Phenol Content (TPC) of Green Lentils

DIC treatments of green lentils were carried out with times between 30 and 240 s and steam pressures between 0.1 and 0.7 MPa. Lentils without any treatment were called the control group. Then, to evaluate the effects of the DIC treatments on the phenolics, flavonoids and antioxidant activity against those of the control, Dunnett’s test was used. Table 1 shows the phenolics, flavonoids and antioxidant activity obtained by the control and DIC samples of green lentils.

First, regarding the total phenolic content (TPC), it can be observed that all the DIC treatments were different from the control. The highest phenolic content was obtained by DIC 11 (0.1 MPa, 135 s) with 0.888 mg eq. gallic acid/g GL, which was 1.47 times higher than that of the control (0.602 mg eq. gallic acid/g GL). On the other hand, the lowest phenolic content was obtained by DIC 13 (0.4 MPa, 135 s) with 0.076 mg eq. gallic acid/g GL. By comparing the obtained results to those of the study of Xu et al. [14], it can be observed that the selected green lentil variety obtained TPC levels below the average of other lentils, such as Red Chief, Crimson, Morton, etc. (~6.96 mg eq. gallic acid/g GL). These differences in results may be attributed to many factors, such as the lentils’ variety, soil, irrigation and climatic conditions, as well as to the type of method used to measure the TPC. It is worth noting that the Folin–Ciocalteu method provides a general approximation of total phenols, but it is not as precise as HPLC, because it can present overestimations due to the presence of pigments such as chlorophyll. In particular, raw GL has been reported to have a total chlorophyll content of approximately 90 μg/g [15]. Therefore, chlorophyll can interfere with the Folin–Ciocalteu reagent, increasing the absorbance value and therefore the estimated content of total phenols in raw GL [16,17,18].

**Figure 1:**
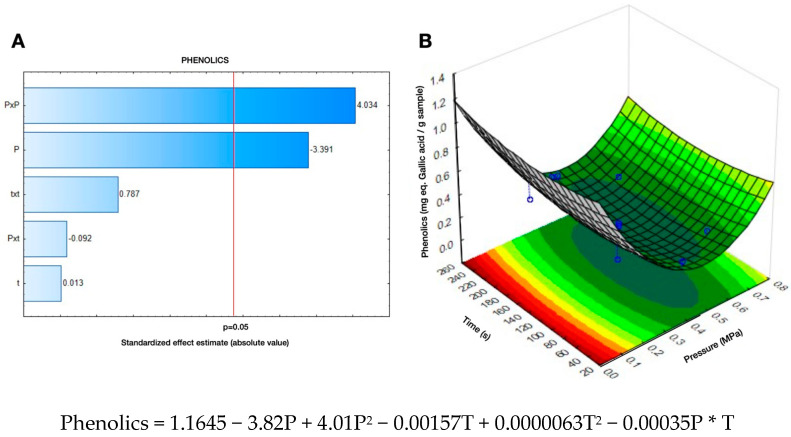
(**A**). Effect of DIC treatment time (s) and steam pressure (MPa) on the total phenol content of green lentils (mg eq. gallic acid/g sample). (**A**) Pareto chart and (**B**) Response surface diagram.

Furthermore, by studying the effect of steam pressure (P) and treatment time (t) on the green lentils’ TPC, it can be remarked that the pressure (P) was a significant variable. As it can be observed in Figure 1A, the Pareto chart (*p* < 0.05) shows that linear (P) and quadratic pressures (P × P) had an impact on the release of phenolic compounds. Furthermore, the response surface diagram (Figure 1B) shows the interactions between the concentration of phenolics (mg eq gallic acid/g GL) and the DIC treatment time (s) and pressure (MPa). In this figure, it can be observed that the greatest amount of available phenolics was obtained under the lowest-pressure treatment (0.1 MPa). Additionally, it can be remarked that under the selected range of treatment times, this variable did not influence phenolics. Therefore, the optimized DIC treatment conditions for maximizing efficiency in the release of phenolics in GL are low pressures (≤0.1 MPa) and short times (~135 s). This phenolic release could be due to the abiotic stress of the food matrix generated by the DIC treatments, which led to the breakdown of the cell wall structure, favoring their availability. This is consistent with the changes in TPC after DIC treatments of grape stem powder and *Hass* avocado seed [13,19].

Moreover, looking at the impact of hydrothermal treatments on polyphenols in lentils, the study of Djabali et al. [20] showed that when measured as pyrocatechol equivalents (PE) per g of dry matter, cooked lentils (100 °C during 45 min) had 72.36 mg PE/g, while raw material had 172.36 mg PE/g. Thus, this study showed that more than half of the initial concentration of phenolic content was transferred to the cooking water. Furthermore, Yeo et al. [21] evaluated the effect of boiling (25 min) on polyphenolic compounds of four varieties of lentils (CDC green land, CDC invincible, 3493-6, and Maxim). This study assessed both the soluble phenolics (including both the free and esterified phenolics) and insoluble-bound phenolics (localized in the cell wall matrices). Their results revealed that boiling triggered a slight increase in soluble phenolics (4.8–8.5%) and an important reduction in insoluble-bound phenolics ~35.6%). According to the literature, hydrothermal treatments weaken the cell wall matrix and release insoluble-bound phenolics [22]. In this respect, DIC as a short thermomechanical treatment may also weaken the cell wall of green lentils and allow for the improved extraction of phenolics. However, to better understand the mechanisms of phenolics’ preservation, further analyses regarding the effects of DIC on both soluble and insoluble-bound phenolics will need to be performed.

### 2.2. Effect of DIC Treatments on Total Flavonoids Content (TFC) of Green Lentils

Table 1 shows that the control obtained the highest TFC value (0.332 mg eq. rutin/g GL) concerning the DIC samples (with ranges between 0.105 and 0.233 mg eq. rutin/g GL). However, by using Dunnett’s test, it can be remarked that DIC 1 (0.4 MPa, 135 s), DIC 2 (0.7 MPa, 135 s), DIC 4 (0.4 MPa, 135 s), DIC 6 (0.61 MPa, 61 s) and DIC 9 (0.19 MPa, 209 s) were not statistically different to the control. Conversely, by comparing the TFC results of the control vs. the lowest value obtained by a DIC treatment, it can be remarked that DIC 10 (0.4 MPa, 135 s) with a TFC of 0.105 mg eq. rutin/g GL triggered around 68% of the TFC losses. The same behavior was found in the study of Djabali, Makhlouf, Ertas and Barkat [20], who after applying a cooking treatment of lentils (100 °C for 45 min), determined a loss of ~59% in TFC for raw material. In that study, the TFC of the raw lentils was 42.82 mg eq. quercitin /g, while the cooked lentils had TFC values of 17.64 mg eq. quercitin/g.

**Figure 2:**
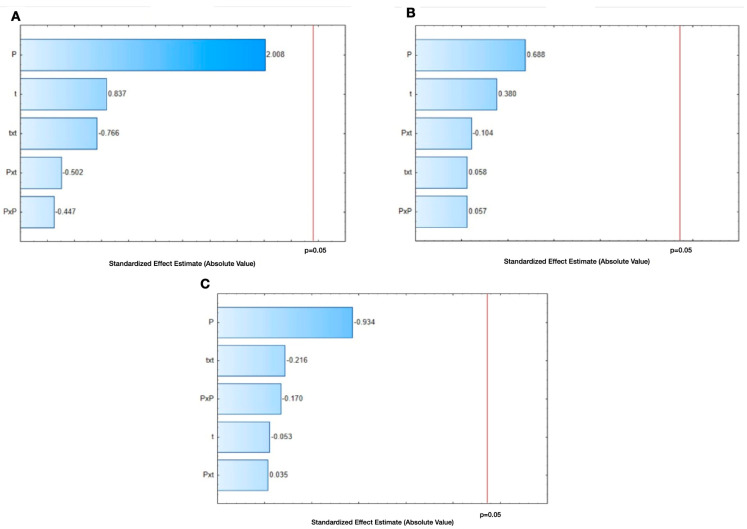
(**A**): Pareto chart of flavonoid concentration expressed as mg eq. rutin/g GL. (**B**): Pareto chart of DPPH expressed as a percentage of discoloration; and (**C**): Pareto chart of TEAC expressed as μM trolox.

On the other hand, as it can be observed in Figure 2A under the selected range of DIC studied parameters, neither the treatment time (t) nor the steam pressure (P) had a significant impact on the TFC of GL. Flavonoids are commonly vulnerable under thermal processing; however, to be consumed, the processing of lentils is inevitable. In this respect, this study showed that by accurately using DIC, it is possible to improve the thermal stability of dietary flavonoids. However, it is still necessary to optimize the DIC parameters to preserve the most important flavonoids, and for that, it is suggested one carries out an HPLC-ESI-MS analysis of green lentils’ flavonoids.

### 2.3. Effect of DIC Treatments on the Antioxidant Activity of Green Lentils

The antioxidant capacity of GL was quantified by DPPH and TEAC assays. A DPPH analysis is based on the interaction of the free radical 2,2-diphenyl-1-picryl-hydrazyl-hydrate with an antioxidant, and the results are expressed as the percentage antioxidant capacity (%) [23]. Table 1 shows the antioxidant capacity of the control and DIC samples of green lentils. First, the results showed that the control obtained an antioxidant capacity of 48.8%, and by using Dunnett’s test, it can be remarked that DIC 5 (0.61 MPa, 209 s), DIC 6 (0.61 MPa, 61 s), DIC 8 (0.19 MPa, 61 s), DIC 9 (0.19 MPa, 209 s), and DIC 12 (0.4 MPa, 30 s) as well as DIC 7, 10 and 13 (0.40 MPa, 135 s) were not statistically different to the control (*p* < 0.05). On the other hand, the rest of the DIC treatments obtained a higher antioxidant capacity than that of the control, with DIC 11 (0.1 MPa, 135 s) being the best treatment with 59.6%. In this respect, some studies have shown that according to the harshness of thermal treatments on food matrices, antioxidant activity can be increased or reduced. For example, after extrusion processing of fiber-enriched lentil flours, the antioxidant capacity was reduced for raw lentils [24]. Conversely, steaming treatments of green peas, yellow peas, chickpeas and lentils resulted in a greater retention of DPPH as compared to boiling treatments [25]. In our study, a Pareto chart (Figure 2B) was performed to elucidate the effect of the steam pressure (P) and the treatment time (t) on the antioxidant capacity of GL. Then, under the selected range of the studied parameters, neither the “t” nor the “P” had a significant impact on the antioxidant capacity of GL.

The DPPH method is one of the most popular assays for the in vitro analysis of complex biological systems, due to its reproducibility, simplicity and velocity. However, the pH and solvent used must be considered, as they can affect the scavenging activity [26]. The enhancement in the antioxidant activity of GL after the DIC treatment could be based on the fact that this process causes the auto-vaporization of the water contained in the GL, triggering an expanded and porous structure that allows for the release of antioxidant compounds.

The TEAC test principle is based on the ability of antioxidants to scavenge 2,2-azinobis (3-ethylbenzothiazoline-6-sulfonic acid) free radical (ABTS^•+^) as compared with a Trolox standard. The ABTS^•+^ radical reacts with a wide variety of antioxidants and includes lipophilic and hydrophilic antioxidant activities [20,21]. The way to interpret the TEAC results is inversely proportional, which means the smaller the TEAC value, the higher the antioxidant activity. The TEAC results of the control and DIC samples are shown in Table 1. First, by using Dunnett’s test, it can be observed that all the DIC treatments were statistically significant compared to the control (427 μM trolox/g GL). Moreover, all the DIC samples showed an improved antioxidant activity, which ranged from 3 to 201 μM trolox/g GL. The best antioxidant activity (<20 μM Trolox/g GL) was obtained by DIC 6 (0.61 MPa and 61 s), DIC 8 (0.19 MPa and 61 s) and DIC 10 (0.4 MPa and 135 s). On the other hand, as shown in Figure 2C, the Pareto chart showed that under the selected range of DIC studied parameters, neither the treatment time (t) nor the steam pressure (P) had a significant impact on the TEAC of GL.

Long processing of legumes generally decreases their antioxidant activity; however, when measuring the antioxidant activity of dry heated beans (*Dolichos lablab* L.), it was remarked that this processing (heating in a microwave oven at 160 °C for 15 min) obtained higher antioxidant activities than those of raw material [27]. According to this study, during this kind of thermal treatment, tannin–protein complexes were formed, and these compounds became potential free radical scavengers and radical sinks. Moreover, Yeo [21] suggested that accurate thermal treatments disintegrate the cell wall matrix of food and allow the bound phenolics to be released. The results indicated that is still necessary to study the mechanism of interaction between thermomechanical treatments and the formation and release of food antioxidants.

### 2.4. High-Performance Liquid Chromatography (HPLC) Analysis of Green Lentil Extracts

The HPLC method was used to separate, identify and quantify polyphenol compounds of GL extracts. Then, since the expected compounds were polar, a non-polar column was used as the stationary phase and polar solvents were used as the mobile phase [28]. Further, to identify and quantify the polyphenol compounds of GL extracts, individual peaks that did not match any of the used standards were expressed as the equivalent of the closest standard. Table 2 shows the results of the HPLC analysis of GL extracts.

First, among all the GL extracts, seventeen peaks were observed, and nine of them were well identified with the standards as gallic acid, dihydroxybenzoic acid, chlorogenic acid, sinapic acid, caffeic acid, syringic acid, 4-hydroxybenzoic acid, vanillic acid and p-coumaric acid. Further, by analyzing the obtained chromatograms, it was possible to highlight that the control extracts obtained the highest concentration of polyphenols with 1317.09 μg/g of GL. Moreover, the control sample showed the widest variety of compounds with nine peaks, identified as gallic acid, dihydroxybenzoic acid, chlorogenic acid and sinapic acid. Moreover, peak 2 with a retention time of 7.342 min was the most abundant polyphenol in the control with 922.8 μg/g of GL (~70% of the total polyphenol concentration). Unfortunately, peak 2 could not be identified, but it was considered to be equivalent to dihydroxybenzoic acid.

Regarding the DIC sample extracts’ results, it can be pointed out that under the selected study’s parameters of steam pressure (P) and treatment time (t), there was an important variation in the polyphenol concentration of each extract, varying from 324.73 to 1256.76 μg/g of GL. DIC 11 (0.1 MPa, 135 s) presented the highest polyphenol concentration compared to the other DIC treatments, and also it performed a greater variety of polyphenols with eight peaks, which were identified as gallic acid, dihydroxybenzoic acid, chlorogenic acid and sinapic acid. This performance was consistent with the TPC results measured by the Folin–Ciocalteu method. In contrast, the lowest polyphenol concentration was obtained by DIC 5 (0.61 MPa, 209 s) with 324.73 μg/g of GL and only two peaks (Peak 2 and syringic acid). Indeed, regarding the polyphenol concentration of DIC 2 (0.7 MPa, 135 s) and DIC 3 (0.7 MPa, 240 s) with 351.27 and 437.94 μg/g of GL, respectively, it can be concluded that the high-steam-pressure treatments reduced significantly the polyphenol concentration. Similar results were found by Yeo and Shahidi [29] who also carried out an HPLC analysis of lentil extracts after boiling and found a significant reduction in phenolics, indicating a possible formation of an irreversible covalent bond between phenolics and other molecules, such as protein, cellulose and starch, due to thermal treatment.

Furthermore, regarding the polyphenol profile of the extracts, it was remarked that peak 2 was the most abundant compound of all the DIC samples with concentrations from 129.09 to 929.51 μg/g of GL, representing an average of 76% of the GL polyphenols. According to Xu and Chang [30], sinapic acid, (+)-catechin and (−)-epicatechin were the most predominant polyphenols of 11 lentil cultivars grown in the United States. However, in this study, sinapic acid was only identified in the control and DIC 6 (0.61 MPa, 61 s), DIC 7 (0.4 MPa, 135 s), DIC 8 (0.19 MPa, 61 s), and DIC 11 (0.1 MPa, 135 s) samples, representing an average of 4.5% of the total polyphenol concentration. These differences could be linked to the seed coat and cotyledon composition variations among lentil cultivars.

In addition, it can be highlighted that some DIC treatments exhibited polyphenol compounds not identified in the control samples or even in other DIC treatments. This was the case with DIC 1 (0.4 MPa, 135 s), which was the only sample that presented caffeic acid (17,767 μg/g). Similarly, syringic acid was present only in the samples in DIC 2 (0.7 MPa, 135 s), DIC 3 (0.7 MPa, 240 s), DIC 5 (0.61 MPa, 209 s), DIC 9 (0.19 MPa, 209 s), DIC 12 (0.4 MPa, 30 s), DIC 4 and DIC 13 (0.4 MPa, 135 s). According to Giusti et al. [31], phenolic acid was also found in Laird and Beluga lentils, but not in dehulled yellow and red lentils. In addition, Ganesan and Xu [8] also stated the presence of syringic acid in lentils; however, they did not specify if the samples were whole or de-husked lentils. Moreover, 4-hydroxybenzoic acid (4HBA) was present in only DIC 8 (0.19 MPa, 61 s), DIC 10 (0.4 MPa, 135 s) and DIC 12 (0.4 MPa, 30 s). According to Irakli et al. [32], 4HBA was the main phenolic acid quantified in five lentil genotypes (*Lens culinaris* L.) produced in Greece. Similarly, the study of Pathiraja et al. [33] showed that 4HBA was mainly found in the seed coats of low-tannin lentil genotypes with a transparent seed coat and yellow cotyledon. What is more, vanillic acid was only found in DIC 12 (0.4 MPa, 30 s), and p-coumaric acid was only present in DIC 13 (0.4 MPa, 135 s). In this respect, previous studies have identified both these compounds in whole and seed-coat lentils [33,34]. In this way, it can be concluded that is necessary to further optimize DIC treatment conditions to better extract phenolic compounds of GL and to conduct a more in-depth study to evaluate the polyphenol profile of green lentils’ coat, as well as whole and de-husked lentils and the DIC’s effect on all samples.

Further, the fact that some DIC treatments exhibited polyphenols that were not present in the control group may be due to the abiotic stress induced by auto-vaporization, which can deform or even break the structure of the cell wall of GL. These damages may generate reactive oxygen species (ROS), causing oxidative stress and probable DNA damage. Then, in response to the stress, a shikimate/phenylpropanoid pathway may be followed, which allows for the biosynthesis of several polyphenols to neutralize ROS [13]. Another explanation is the fact that phenolic compounds in legume seeds interact with their cell walls. Particularly, phenols have a high affinity for dietary fiber elements, such as pectin and cellulose, due to their covalent bonds, hydrogen bonds, and hydrophobic interactions [35,36]. Thus, it could be possible that DIC treatment reduces these linkages and, in this way, increases the availability of phenolic compounds [37]. A similar behavior was found in grape pomace treated with DIC, in which the extraction of phenolic compounds was improved thanks to the weakening of the hydrophobic interactions and hydrogen bonds between the phenols and the cell wall polysaccharides [38].

## 3. Materials and Methods

The green lentils (GL) were obtained from the French store “*Carrefour”* under the brand *Vivien Paille* (France). They were kept in a cool, dry place before any treatment. For the chemical analysis, all the standards and solvents were purchased from Sigma-Aldrich (Sigma-Aldrich, St. Louis, MO, USA).

### 3.1. Instant Controlled Pressure Drop (DIC)

DIC treatments were executed using DIC-MP equipment (ABCAR-DIC Process, La Rochelle, France) following a central composite rotatable experimental design (Table 3). This design yielded 13 treatments with four factorial points (+1, +1; +1, −1; −1, −1; −1, +1), five central points (0.0) and four star points (+α, 0; 0, +α; −α, 0; 0, −α). DIC treatment applied on lentils consisted of the following: (a) placement of 100 g of lentils into the reactor under atmospheric pressure; (b) establishment of initial vacuum (~10 kPa); (c) injection of saturated steam (ranging from 0.1 to 0.7 MPa); (d) holding of target saturated steam pressure (treatment time ranging from 30 to 240 s); (e) instant controlled pressure drop towards the vacuum (between 5 and 10 kPa); (f) holding of vacuum (~5 s); and (g) release of samples at atmospheric pressure. Table 3 describes the DIC treatments for the conditions studied. After DIC treatment, a post-drying stage was carried out in a convective air drier (Memmert D06064UNB 800 Model, Schwabach, Germany) at 50 °C with an airflow of 1 m/s for 24 h.

### 3.2. Extracts Preparation

DIC-treated lentil samples and the control (raw material) were ground with a coffee grinder (Krups GX4100, Solingen, Germany) until a fine powder was obtained. After that, 0.5 g of each sample was weighed in a 50 mL conical plastic centrifuge test tube and mixed with 10 mL of ethanol acidified with 1% hydrochloric acid. Subsequently, at room temperature (25 °C), samples were left for 2 h under orbital agitation (Junior orbit shaker 3520, Chennai, India), covered from light, at 130 rpm. After agitation, the samples were centrifuged (Hermle Z 383 K, Wehingen, Germany) at 7265 g for 10 min. Finally, the supernatant was recovered and stored at −20 °C for future analyses. All samples were prepared in triplicate.

### 3.3. Total Phenolics Content

To determine the phenolic content, the colorimetric Folin–Ciocalteu method was used. This method is based on the reduction in the Folin reagent, passing from an oxidation state of 6^+^ to 5^+^, and such reduction is observed when the color changes from yellow to blue [39,40]. Then, to evaluate the total phenol content of green lentils, a gallic acid calibration curve was made with values from 0 to 1000 µM. The samples were analyzed in triplicate in a 96-well plate. A total of 20 µL of the sample was added to each well, followed by 150 µL of water and 50 µL of Folin–Ciocalteu reagent (5N). The plate was protected from light for 5–8 min. After this time, 50 µL Na_2_CO_3_ (20% *w*/*v*) was added and protected from light for 2 h. The plate was read with xMark™ Microplate Absorbance Spectrophotometer (Bio-Rad, Tokyo, Japan) at 760–765 nm. Results were expressed as mg of gallic acid/g of GL.

### 3.4. Antioxidant Capacity by 2,2-Diphenyl-1-picrylhydrazyl (DPPH)

To determine antioxidant capacity, the Fukumoto and Mazza [41] method was used. In a 96-well plate, 20 µL of the sample was added to each well, followed by 200 µL DPPH (125 µM). To enable the chemical reaction, the plate was left for 90 min at room temperature (25 °C), protected from light. The plate was read with xMark™ Microplate Absorbance Spectrophotometer (Bio-Rad, Japan) at 520 nm. Antioxidant capacity was expressed as the percentage of discoloration of the DPPH without a sample. All samples were analyzed in triplicate.

### 3.5. Trolox Equivalent Antioxidant Capacity (TEAC)

The cationic free radical 2,2′-Azino-bis (3-ethylbenzothiazoline-6-sulfonic acid) (ABTS) was used to measure antioxidant capacity. A Trolox calibration curve was made with values from 0 to 700 µM. To generate the free radical, potassium persulfate (2.45 mM) and ABTS (7 mM) protected from light were mixed for 16 h at room temperature. The solution was diluted with ethanol, until reaching an approximate absorbance of 0.8, which was read at 734 nm with xMark™ Microplate Absorbance Spectrophotometer (Bio-Rad, Japan). Then, to evaluate the Trolox equivalent antioxidant capacity of lentils, 200 µL of the ABTS radical and 20 µL of GL were added to each well. To enable the chemical reaction, the plate was kept under continuous stirring for 6 min, and later it was read at 734 nm [42]. Each sample was analyzed in triplicate.

### 3.6. Total Flavonoids Content

The sample extracts were analyzed in triplicate in a 96-well plate. A total of 50 µL of sample, 180 µL distilled water and 20 µL of 2-aminoethyl diphenylborinate (10 g/L) were added to each well. The plate was read with xMark™ Microplate Absorbance Spectrophotometer (Bio-Rad, Japan) at 404 nm. A rutin calibration curve was made with values from 0 to 500 µg/mL. Flavonoid content was expressed as mg eq rutin/g of GL [43].

### 3.7. Phenolic Content by HPLC

Lentils’ extracts and mobile phases were filtered through a 0.45 μm nylon membrane filter. HPLC analysis was performed using an Agilent 1200 HPLC system (Agilent Technology 1200 series, Palo Alto, CA, USA), equipped with a quaternary pump, autosampler and a diode array detector. Separation of phenolics compounds was performed using an Agilent Eclipse XDB-C18 column (5 μm, 4.6 mm, 150 mm) at 25 °C. The mobile phase consisted of phosphoric acid 0.01 M (A) and pure methanol (B) at a flow rate of 0.7 mL/min. The linear gradient conditions were 0–25 min 5% A, 25–50 min 40% A, 50–65 min 50% A, 65–70 min 70% A, 70–80 min 80% A and 80–85 min 100% A, with a total time of 85 min. The standards used were as follows: gallic acid, dihydroxybenzoic acid, 4-Hydroxybenzoic acid, catechin, chlorogenic acid, vinylic acid, caffeic acid, syringic acid, p-coumaric acid, 4-Hydroxy-3-methoxycinnamic acid, sinapic acid, trans 2-hydroxycinnamic acid and apigenin. Standards were prepared in methanol, and concentrations were the same for all standards (0.007 to 0.038 mg/mL) [44].

### 3.8. Statistic Analysis

The statistical analysis was performed in STATISTICA software, version 12. Two statistical analyses were carried out, and a *p*-value less than 0.05 was taken as statisti-cally significant. The first was a two-way ANOVA, followed by a Pareto chart and, if the result was statistically significant, a response surface diagram, to determine which of the DIC conditions (time, pressure and/or their interaction) was responsible for modifying the evaluated parameters (concentration of phenolics and flavonoids as well as the antioxidant activity). The second was to compare the results of the DIC treatments in terms of phenolics, flavonoids, and antioxidant capacity by 2,2-diphenyl-1-picrylhydrazyl (DPPH) and Trolox equivalent antioxidant capacity (TEAC) against those of the control (Dunnett’s test, *p* < 0.05). 

## 4. Conclusions

Lentils are mainly cooked by boiling, which causes the loss of antioxidant compounds and reduces their antioxidant activity. For this reason, DIC is a promising emerging technology to preserve the macro- and micronutrients of lentils. In this respect, this study shows that through DIC treatments of green lentils, it was possible to promote the release of their phenolic compounds and maintain their antioxidant capacity. The most efficient conditions were found under low pressures (<0.1 MPa) and short times (<160 s). Moreover, to optimize DIC treatment, it is necessary to conduct a more in-depth study to evaluate the polyphenol profile of green lentils’ coat, as well as whole and de-husked lentils. Further, to elucidate how thermomechanical treatments improve TEAC antioxidant activity, it will be necessary to evaluate the evolution of lentils’ matrices after DIC treatment. Finally, another factor to take into account for new lentil food products is the content of non-nutritional compounds in lentils, such as phytates and oligosaccharides. The optimization of DIC treatments by taking into account all these variables will allow us to achieve a high-quality food product.

## Figures and Tables

**Table 1:** Phenolics, flavonoids and antioxidant activity of green lentils.

Treatment	Pressure (MPa)	Time (s)	Phenolics (mg eq. Gallic Acid/g GL)	Flavonoids (mg eq. Rutin/g GL)	DPPH (%)	TEAC (μM Trolox/g GL)
Control	-	-	0.602	0.332	48.8	427
DIC 1	0.40	135	0.138 *	0.173	53.8 *	201 *
DIC 2	0.70	135	0.285 *	0.218	56.8 *	41 *
DIC 3	0.70	240	0.309 *	0.110 *	56.3 *	82 *
DIC 4	0.40	135	0.221 *	0.233	52.8 *	102 *
DIC 5	0.61	209	0.226 *	0.144 *	45.6	45 *
DIC 6	0.61	61	0.252 *	0.229	47.6	3 *
DIC 7	0.40	135	0.245 *	0.113 *	46.6	53 *
DIC 8	0.19	61	0.375 *	0.128 *	47.5	7 *
DIC 9	0.19	209	0.372 *	0.189	46.6	44 *
DIC 10	0.40	135	0.127 *	0.105 *	49.1	13 *
DIC 11	0.10	135	0.888 *	0.133 *	59.6 *	191 *
DIC 12	0.40	30	0.285 *	0.133 *	50.6	146 *
DIC 13	0.40	135	0.076 *	0.129 *	47.3	44 *

* Means statistically different from the control (Dunnett, *p* < 0.05). The results are the average of triplicates. GL: green lentil; DPPH: antioxidant capacity in percent discoloration; TEAC: Trolox equivalent antioxidant capacity.

**Table 2:** Identification and quantification of polyphenols in the GL extract by high-performance liquid chromatography (HPLC).

Compound	RT	Control	DIC 1	DIC 2	DIC 3	DIC 4	DIC 5	DIC 6	DIC 7	DIC 8	DIC 9	DIC 10	DIC 11	DIC 12	DIC 13
Steam pressure (MPa)		NA	0.4	0.7	0.7	0.4	0.61	0.61	0.4	0.19	0.19	0.4	0.1	0.4	0.4
Treatment time (s)		NA	135	135	240	135	209	61	135	61	209	135	135	30	135
Gallic acid	5.584	13.62								15.50		16.53	20.77	19.04	14.49
Peak 2	7.342	922.8	454.01	319.36	391.91	599.01	284.94	443.97	457.93	742.17	538.58	739.04	129.09	929.51	511.53
Dihydroxybenzoic acid	9.62	93.10	19.13			19.18		21.97	22.90	59.14	31.009	28.26	84.28	44.2	24.75
Peak 4	9.993	41.69	20.17												
Peak 5	13.09												34.94		
4-Hydroxybenzoic acid	13.10									31.65		24.74		36.54	
Peak 7	14.95										53.68				
Chlorogenic acid	15.57	63.5				23.17		24.50	22.71	40.37		30.97	50.85		25.78
Vanillic acid	16.991													211.08	
Caffeic acid	17.76		34.29												
Syringic acid	18.95			31.91	46.03	89.49	39.79				99.338			16.39	105.25
Peak 12	19.08	111.8						40.10	54.75	106.51		124.11	115.56		
Peak 13	21.86	11.78											10.22		
p-coumaric acid	22.47														8.63
Peak 15	23.42											25.39			
Synapic acid	24.86	46.12						25.94	24.29	34.29			33.81		
Peak 17	44.85	12.60													
Total peaks	17	9	4	2	2	4	2	5	5	7	4	7	8	6	6
Total concentration		1317.09	527.6	351.27	437.94	730.85	324.73	556.48	582.58	1029.63	722.607	989.04	479.52	1256.76	690.43

Concentrations expressed in μg/g of GL. DIC: instant controlled pressure drop; RT: retention time (min).

**Table 3:** Central composite and experimental design for the DIC treatment of green lentils.

Points	Pressure (MPa)	Time (s)
Point min (−α)	0.10	30
Point (−1)	0.19	61
Central point	0.40	135
Point (+1)	0.61	209
Point max (+α)	0.70	240
Value of 1	0.21	74
**DIC treatments**	**Pressure (MPa)**	**Time (s)**
1	0.4	135
2	0.7	135
3	0.7	240
4	0.4	135
5	0.61	209
6	0.61	61
7	0.4	135
8	0.19	61
9	0.19	209
10	0.4	135
11	0.1	135
12	0.4	30
13	0.4	135

## Data Availability

All related data and methods are presented in this paper. Additional inquiries should be addressed to the corresponding author.

## Sample Availability

Not available.
